# Gene Expression Programs of Mouse Endothelial Cells in Kidney Development and Disease

**DOI:** 10.1371/journal.pone.0012034

**Published:** 2010-08-10

**Authors:** Eric W. Brunskill, S. Steven Potter

**Affiliations:** Division of Developmental Biology, Children's Hospital Medical Center, Cincinnati, Ohio, United States of America; University of Barcelona, Spain

## Abstract

Endothelial cells are remarkably heterogeneous in both morphology and function, and they play critical roles in the formation of multiple organ systems. In addition endothelial cell dysfunction can contribute to disease processes, including diabetic nephropathy, which is a leading cause of end stage renal disease. In this report we define the comprehensive gene expression programs of multiple types of kidney endothelial cells, and analyze the differences that distinguish them. Endothelial cells were purified from *Tie2-GFP* mice by cell dissociation and fluorescent activated cell sorting. Microarrays were then used to provide a global, quantitative and sensitive measure of gene expression levels. We examined renal endothelial cells from the embryo and from the adult glomerulus, cortex and medulla compartments, as well as the glomerular endothelial cells of the *db/db* mutant mouse, which represents a model for human diabetic nephropathy. The results identified the growth factors, receptors and transcription factors expressed by these multiple endothelial cell types. Biological processes and molecular pathways were characterized in exquisite detail. Cell type specific gene expression patterns were defined, finding novel molecular markers and providing a better understanding of compartmental distinctions. Further, analysis of enriched, evolutionarily conserved transcription factor binding sites in the promoters of co-activated genes begins to define the genetic regulatory network of renal endothelial cell formation. Finally, the gene expression differences associated with diabetic nephropathy were defined, providing a global view of both the pathogenic and protective pathways activated. These studies provide a rich resource to facilitate further investigations of endothelial cell functions in kidney development, adult compartments, and disease.

## Introduction

Endothelial cells play essential roles in both development and disease. During development there is a crucial cross talk with surrounding tissues. Heterotopic transplantation studies show that organ specific microenvironments drive the specificity of vasculature formed. For example peripheral blood vessels that penetrate a graft of brain tissue form tight junctions, typical of brain vessels [1]. Conversely, signals from endothelial cells have been shown to be essential during the development of multiple organ systems, including the heart [2], pancreas [3], liver [4], and kidneys [5]. There exist a tremendous variety of endothelial cell types, and we are only beginning to understand their diverse functions [6].

Endothelial cell dysfunction can also play a primary role in disease, including diabetic nephropathy [7]. Type 2 diabetes is an increasingly important global health threat. In the United States the prevalence of type 2 diabetes has almost doubled in the past 25 years, and in Asia the rate of increase is even more dramatic [8]. Diabetes is now the most common cause of end stage renal disease in both developed and emerging nations [9]. All three cell types of the glomerulus have been strongly implicated in diabetic nephropathy. The mesangial cells produce the observed mesangial matrix expansion. Altered podocyte function, including podocyte loss, foot process effacement, and altered makeup of the glomerular basement membrane (GBM), result in increased protein leakage. In addition altered endothelial cell function has been associated with increased leukocyte recruitment [10], increased angiogenesis leading to the formation of immature and leaky vessels [11], and decreased production of activated protein C, which normally inhibits podocyte and endothelial cell apoptosis [12].

Important insight into both disease and normal developmental processes can be gained by gene expression profiling. Microarrays provide a comprehensive, sensitive and quantitative measure of gene expression. Their global readout of gene use gives a detailed picture of expressed transcription factors, growth factors, and receptors. Early pioneering studies used microarrays to examine changing gene expression patterns of entire kidneys from the rat as a function of developmental time [13], followed by similar work with mouse [13], [14]. In some cases it was possible to use manual microdissection or FACS to define gene expression profiles of selected specific structures or cell types [14], [15], [16]. We have previously described a gene expression atlas of kidney development, at microanatomic resolution [17]. We primarily used laser capture microdissection to isolate most of the multiple components of the developing kidney. Microarrays were then used to define gene expression patterns. The results defined the changing waves of gene usage as a function of nephrogenesis. In addition, novel molecular markers of specific compartments were found. Further, by examining the concordance of changing transcription factor expression with the presence of evolutionarily conserved transcription factor binding sites within the promoters of activated genes it was possible to begin to generate a genetic regulatory network of kidney development.

In this study we extend this previous work by investigating the gene expression programs of kidney endothelial cells. In particular, we first defined the gene expression profile of endothelial cells from E15.5 embryonic kidneys, to better understand the genetic program that drives the formation of the renal vascular system. We also examined the gene expression patterns of the adult glomerular, medullary, and cortical endothelial cells, to discover the molecular basis of their compartment specific properties. Finally, to better define the molecular anatomy of diabetic nephropathy, we analyzed the altered gene expression patterns of glomerular endothelial cells in *db/db* mutant mice, which represent a useful model of this disease.

## Results and Discussion

### Gene expression profile comparisons

We used *Tie2-GFP* transgenic mice to purify endothelial cells from E15.5 embryonic kidneys, adult medulla, adult glomeruli, adult cortex (with glomeruli removed), and adult glomeruli of *db/db* mice, which, again, represent a mouse model for diabetic nephropathy. Glomeruli were purified by sieving. Tissues were rapidly dissociated using a combination of enzymatic digestion and trituration, followed by FACS isolation of GFP positive cells. Affymetrix Mouse Gene 1.0 ST arrays were used to provide global, sensitive and quantitative measures of gene expression. In each case at least three independent biological samples were examined. The results yield a universal view of the gene expression programs used by these distinct types of endothelial cells.

**Figure 1:**
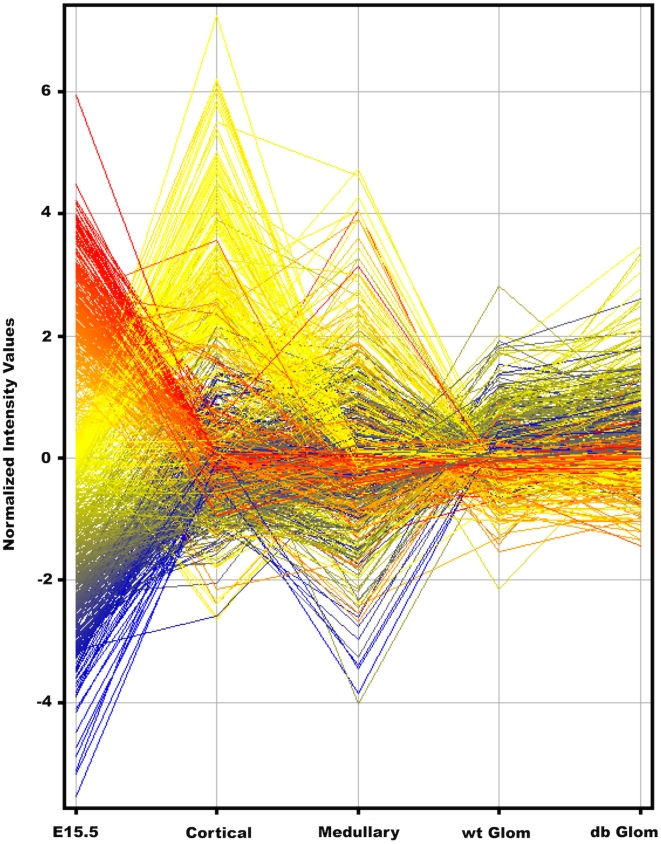
Profile plot of gene expression differences of renal endothelial cells isolated from different developmental times, compartments, and disease states. 786 probesets, each represented by a single line, color coded according to expression in embryonic cells, with red indicating high and blue representing low levels of expression. **E15.5**, total E15.5 kidney endothelial cell population, **Cortical**, endothelial cells from adult kidney cortex, with glomeruli removed, **Medullary**, adult medulla compartment endothelial cells, **wt Glom**, control adult glomerulus compartment endothelial cells, **db Glom**, adult glomerulus compartment endothelial cells from *db/db* mouse with diabetic nephropathy.

A profile plot provides an overview of the observed differences in gene expression (Fig. 1). The array data was analyzed with GeneSpring GX11.01, RMA normalized, and only probesets with a minimum 100 raw expression level in three samples were included. Following ANOVA analysis, P<0.05, we selected for a minimum five fold difference in expression level in any pairwise sample type comparison, thereby identifying 786 probesets with the most distinct expression patterns (Fig. 1)(Supplementary Data S1). The embryonic endothelial cells showed the most distinct gene expression signature, while the adult glomerular endothelial cells from normal and *db/db* mice were most similar.

### Embryonic endothelial cells

We first compared embryonic to adult endothelial cell gene expression profiles, to better define the gene expression program of renal vascular development. Screening for genes with at least four fold difference in expression between E15.5 and normal adult (glomerular, cortical and medullary) endothelial cells and further requiring higher expression in embryonic samples, to focus on genes driving the embryonic endothelial program, identified 340 probe sets (Supplementary Data S2). Gene ontology biological process analysis, using ToppGene, identified forty processes with P values listed as zero, and all were related to cell division (Supplemental Data S3). They included, for example, M phase, cell cycle, mitosis, nuclear division, cell division, DNA replication, regulation of cell cycle, and so on. Not unexpectedly, the results showed clearly that the embryonic endothelial cells are heavily committed to cell division, while adult cells are not.

### Embryonic endothelial cells compared to other embryonic compartments

**Figure 2:**
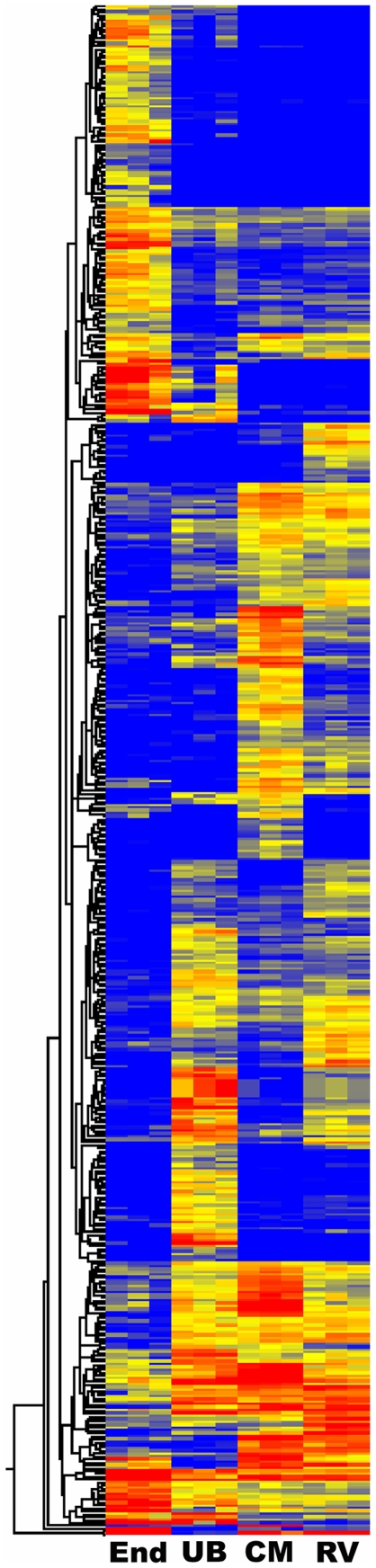
Heat Map displaying gene expression differences (531 probe sets) between different kidney development compartments. Shown are **End** (E15.5 endothelial cells), **UB** (E11.5 ureteric bud), **CM** (P0 capping mesenchyme), and **RV** (P4 renal vesicle).

It is perhaps more instructive to compare the gene expression profile of the embryonic endothelial cells to those of other embryonic compartments, thereby identifying those genes specific to vasculature formation. We therefore selected renal vesicles, capping mesenchyme and ureteric bud for comparison. An ANOVA analysis, including Benjamini and Hochberg correction, corrected P<0.02, and fold change of at least three, gave 531 genes with strong expression differences in the four compartments. A visual representation of the distinctive gene expression signatures of these four compartments is shown in Fig. 2.

To capture a broader view of the genetic basis of embryonic vasculogenesis we also performed a less stringent analysis, requiring only a minimum two fold change (instead of three). We focused on genes with elevated expression in endothelial cells in all pairwise comparisons, yielding a total of 207 probe sets (Supplemental Data S4), which were then subjected to functional annotation using GeneSpring. The top biological processes to emerge included angiogenesis (*Elk3, Epas1, Egfl7, Eng, Sox18, Kdr, Flt1, Robo4, Anxa2),* regulation of cell migration *(Abi3, Pecam1, Egfl7, Tek, Tie2* and *Robo4*), and VEGF signaling pathway (*Flt4, Kdr, Flt1*). Many of the genes on this list of 207, including *Mef2c*, *Ets1, endoglin, Fli1, Tie1, Flt1, Cdh5,Fflt4, Sox18, Erg, Epas1, Ahr, Pecam1*, and *Icam2*, have been previously strongly implicated in endothelial cell development [18], providing historic validation of the screen.

**Figure 3:**
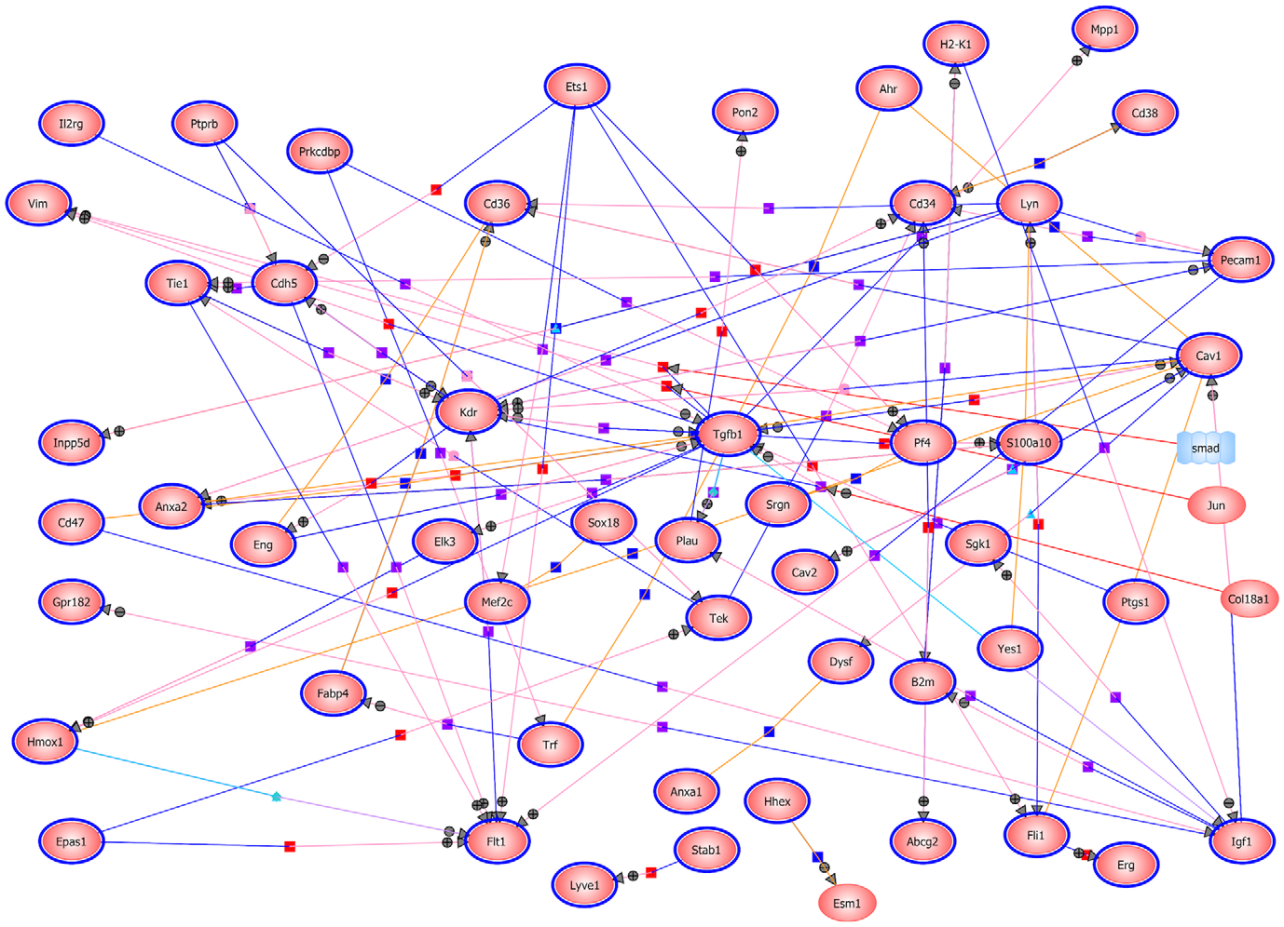
Molecular pathways analysis of genes with elevated expression in the kidney developing endothelial compartment. Generated with GeneSpring 11.0 using the 207 genes with elevated expression in the embryonic endothelial cells. Relations between encoded proteins are shape and color coded, with dark purple square  =  member, light blue triangle  =  transport, red square  =  expression, purple square  =  regulation, blue square  =  binding, orange circle  =  promoter binding, light blue diamond  =  metabolism, pink  =  protein modification.

The expression data was further examined by performing a molecular pathways analysis using GeneSpring. The results showed a number of interesting interactions, with *Tgfbeta1* and *Kdr* occupying central positions (Fig. 3). *Kdr* encodes a tyrosine kinase receptor for VEGF, and has been previously shown to mediate endothelial cell proliferation, survival, migration, tubular morphogenesis and sprouting [19]. Tgfbeta1 is also a well-established modulator of angiogenesis [19].

These 207 genes can be divided into functional categories, with DAVID identifying, for example, a group of 15 transcription factors (*Epas1, Nfib, Elk3, Erg, Fli1, Klf7, Ets1, Hhex, Sox18, Rnf141, Bcl6b, Elf4, Ahr, Ostf1, Mefc2*)(Supplemental Data S5). It is interesting that three of these genes, *Hhex*, *Bcl6b* and *Elf4*, have been shown to be involved in hematopoiesis [20], [21], [22]. This dual endothelial-hematopoetic character strongly indicates the presence of hemangioblasts, multipotent precursors able to differentiate into both endothelial and hamatopoetic cells, in the E15.5 embryonic kidney, as has been previously reported [23]. In addition, this analysis identified 60 genes encoding membrane/receptor proteins, many with previously established function in the development and function of endothelial cells, as well as other functional groups of genes.

A ToppGene functional analysis of the list of 207 genes with elevated expression in embryonic endothelial cells gave similar results, providing lists of genes involved in angiogenesis, vasculogenesis, blood vessel morphogenesis, response to oxygen levels, hemopoiesis, and other biological processes these cells are engaged in (Supplementary Data S6). In addition the ToppGene analysis identified key evolutionarily conserved transcription factor binding sites (RGAGGAARY-V$PU1_Q6, V$STAT6_02, V$STAT4_01, V$ELF1_Q6, RYTTCCTG_V$ETS2_B and V$ETS2_B) enriched in the proximal promoter regions of multiple genes with elevated expression (Supplementary Data S6). For example conserved RYTTCCTG_V$ETS2_B binding sites were found upstream of 25 genes (*Egfl7*, *Rasgrp3*, *Tll1, Erg, Sgk1, Adpr, Prkch, Myct1, Fxyd5, Vamp5, Ets1, Hhex, Sipa1, Lyn, ldb2, Gpr183, Adcy4, Igf1, Mef2c, lcp1, Flt1, Elk3, Gpr97, Cdh5, Robo4*). It is particularly interesting that ETS binding sites emerge with extremely high statistical significance, given the known vital role of ETS transcription factors in vasculogenesis [24]. STAT binding sites were also found with great frequency, again of interest because STAT3 has also been shown to be an important regulator of genes involved in vasculogenesis [25], [26]. Of interest, STAT3 is expressed in the embryonic endothelial cells, but also in the other endothelial cells, so it is not on the list of 207 genes with up regulation in embryos. Its activity, of course, is regulated by Jak mediated phosphorylation. These results begin to define a genetic regulatory network driving development of the renal vasculature.

### Glomerular specific endothelial genes

The glomerular endothelial cells (GEC) are striking in their fenestrated structure, which facilitates fluid bypass to the glomerular basement membrane. To better define the gene expression signature specific to the adult GEC we compared their gene expression profile to those of kidney medullary and cortical (with glomeruli removed) enthothelial cells. The microarray data screen included subtraction of low expressing genes, where raw signal was not above at least 100 for three individual samples, an ANOVA analysis, P<0.05, and requiring at least a four fold elevated expression in endothelial cells of glomeruli compared to both cortical and medullary endothelia. This stringent screen identified twelve genes.

*Scn7a* showed the greatest fold change, with more than ten fold elevated expression in GEC compared to both cortex (with glomeruli removed) and medullary endothelial cells. This gene encodes a membrane bound voltage gated member of the sodium channel family. Endothelial cells are generally considered to be electrically unexcitable, although there have been reports that that this is not always the case [27], [28]. In particular, it was shown that in hamster feed arteries electrical stimulation of endothelial cells can result in a vasodilation reponse [27]. This suggests the possible presence of a novel electrical signaling pathway in the glomerulus.

The microarray data also showed the *Ehd3* gene to be strongly up regulated in GEC. *Ehd3* encodes a protein involved in endosome to golgi transport [29]. Of interest, this gene has been previously shown to be expressed very specifically in the GEC, providing additional historical validation of the microarray data [30]. Indeed, this was the only gene, to our knowledge, previously shown to be expressed by GEC and not other endothelial cells of the kidney.

Another gene up regulated in GEC was *Gata5*, encoding a transcription factor previously shown to be required for the differentiation of cardiac endothelial cells [31]. We also observed a dramatic up regulation of *Sema5a*, which has been shown to play a critical role in branching patterning of the vascular system [31]. It has also been shown to promote endothelial cell proliferation and to protect against apoptosis [32]. Of further interest, the SEMA5A protein includes seven thrombospondin repeats.

We also found elevated GEC expression of the *Adamts5* gene, which encodes a disintegrin like and metalloprotease with thrombospondin domains. This gene has previously been shown to be expressed in the endothelial cells of the adult cornea [33]. The first thrombospondin repeat, but not the second, functions as an inhibitor of angiogenesis [34]. Another GEC elevated gene, *Gimap3*, encodes a GTPase previously implicated in the control of cell survival [35].

*Nostrin* also showed much higher expression in GEC than other endothelial cells of the kidney. It encodes a protein that binds the enzyme responsible for NO synthesis, endothelial NO synthase, and triggers its translocation from the plasma membrane to vesicle-like subcellular structures, thus attenuating NO production. For a complete list of genes up regulated in GEC see Supplementary Data S7.

It is perhaps surprising that so few genes are strongly GEC specific in expression. In this regard it is interesting to note that the formation of fenestrae has been clearly linked to VEGF signaling, PI3 kinase activation and actin rearrangements [36], in a pathway that might involve relatively little *de novo* gene expression.

### Medullary specific endothelial genes

The renal medulla presents extreme conditions, with low oxygen tension and high concentrations of the solutes NaCl and urea in antidiuresis. The cells of the medulla employ a variety of mechanisms to survive this hostile environment [37]. We specifically examined the gene expression profile of the medullary endothelial cells in comparison to the glomerulus and cortex. Using the same microarray data screening procedure described for the glomerular compartment, only requiring five fold upregulation instead of four, fourteen genes were identified with elevated expression in medullary endothelial cells, as described below.

Insulin-like growth factor 1 (*Igf1*) expression was the most specific to medullary endothelial cells, elevated 15 fold and 28 fold compared to cortical and glomerular compartments, respectively. *Igf1* is implicated in neovasculartization [38], migration and angiogenesis of endothelial cells [39]. Of interest, it induces hypoxia-inducible factor 1 mediated VEGF expression in colon cancer cells [40]. Other elevated genes were *Tll1*, required for normal heart development and vasculogenesis [41], and *Tmem195*, encoding a transmembrane protein. In addition there was medullary endothelial cell specific expression of the *Cd36* receptor gene, whose encoded protein interacts with a large variety of ligands, including collagen types I and IV, thrombospondin, platelet-agglutinating protein p37, oxidized low density lipoprotein, and long-chain fatty acids.

It is interesting to note that *Pde7b* and *Agtrl1* both normally showed restricted expression in the medullary endothelial cells, but were observed to be upregulated in the diabetic nephropathy GEC, as discussed later.

Other genes with medullary elevated expression included *Mmp-11*, encoding a matrix metalloproteinase, previously reported localized surrounding endothelial cells of glioblastomas [42], We also observed elevated medullary endothelial cell expression of *Angpt2*, which is normally expressed at sites of vascular remodeling, and functions as an antagonist of the angiogenic functions of angiopoietin1 and TIE2 [43]. See Supplementary Data S8 for a complete gene list.

### Cortical endothelial cells

Of interest, while relatively few genes showed glomerular or medullary endothelial cell specific expression with the stringent screen employed, a similar screen for cortical endothelial cell specific gene expression resulted in a much larger number, 125 genes (see Supplemental Data S9). Further analysis of this gene set gave a surprising result, with the gene expression signature resembling that previously described for proximal tubules [17]. For example, there were twenty five transporter genes, with almost all previously shown to have very strong proximal tubule specific expression (GUDMAP.org). The data, therefore, indicated that there was a minor contamination of the cortical endothelial cells and that when the expression patterns of the medulla and glomerular endothelial cells were subtracted the remainder was primarily a proximal tubule profile. Even a small contamination would be revealed by the comparative analysis performed, with genes showing high expression in contaminating cells showing up when the endothelial gene expression pattern was removed. Nevertheless, the cortical endothelial data set was useful for the purpose of screening the glomerular and medulla profiles for compartment specific gene expression. It served to subtract cortical endothelial expressed genes, even though some additional proximal tubule expressed genes were also represented.

We examined the other data sets for signs of similar contamination. For example GEC might be particularly prone to contamination with podocytes and mesangial cells, the remaining two major cell types of the glomerulus. Other cell varieties would be effectively removed by the initial sieving procedure used to purify the glomeruli. Two specific markers of podocytes and mesangial cells are *Mafb* and *Meis1*, respectively. These genes showed low expression levels in the GEC samples, indicating a high level of purity.

### Comparison of glomerular endothelial cells of normal and *db/db* mice

**Figure 4:**
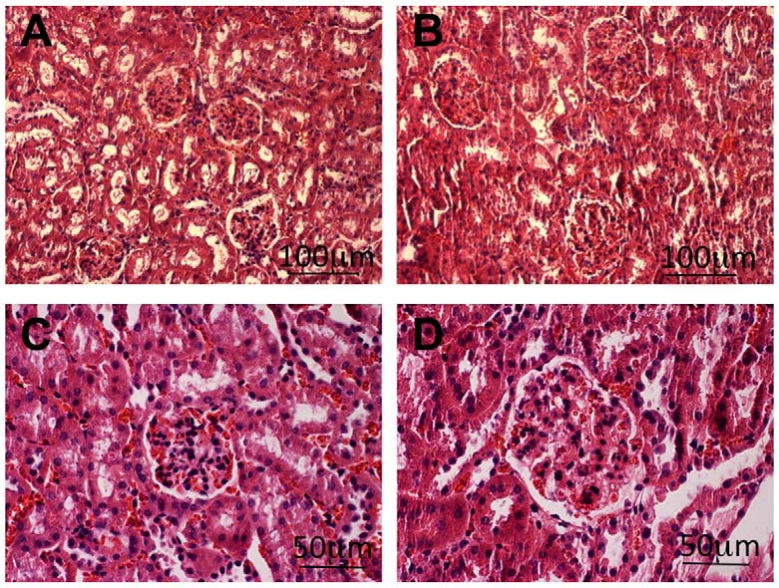
Enlarged glomeruli in seven month old *db/db* mice. Panels A and C are controls, and panels B and D are from *db/db* mice. Panels A and B are lower magnification views showing multiple glomeruli, while panels C and D are higher magnification, more clearly showing the size difference. Sections were hematoxylin and eosin stained.

To better understand the molecular basis of diabetic nephropathy we compared the wild type and *db/db* GEC gene expression profiles. The *db/db* mice, with loss of function of the leptin receptor gene, suffer obesity, diabetes, and diabetic nephropathy. The *db/db* mice show many similarities to human diabetic nephropathy, including elevated glucose levels, glomerular hypertrophy, mesangial matrix expansion and albuminuria [44], [45]. We evaluated the *db/db* mice when seven months of age, at which point they exhibited profound obesity (63.1±2.6 grams compared to 39.1±2.4 grams for non *db/db* littermates), elevated blood sugar (7302±5258 mg/dL versus 47±4.2 for controls), and albuminuria (3911±1288.5 µg/ml/24 hr versus 32.3±5.3 for controls). At this point the glomeruli showed significant enlargement (Fig. 4). We analyzed the relatively early stages of the disease process to better understand the initiating events. The glomeruli were, as before, first purified by sieving, followed by cell dissociation and FACS, to isolate the *Tie2-GFP* positive endothelial cells.

We initially used a relatively low stringency screen of the resulting microarray data, performing an unpaired t-test, P<0.05, finding a total of 267 probesets with greater than 1.5 fold change in expression (Supplemental Data S10). ToppGene was then used for functional analysis, with the following interesting results.

The three gene ontology molecular functions listed with the lowest P-values were all related to phosphodiesterase activity (P = 0.0001–0.0002), suggesting altered cAMP levels in the *db/db* endothelial cells. The biological processes that emerged with lowest P values were cell adhesion and biological adhesion, with 26 genes each (P = 0.00003). Additional biological processes identified were response to external stimulus, cellular extravasation, heterophilic cell adhesion, cell adhesion, biological adhesion, leukocyte adhesion, inflammatory response, and leukocyte tethering and rolling. ToppGene also identified several conserved transcription factor binding sites within the promoters of genes with altered expression, including V$STST5B_01 upstream of *Klf4*, *Pcolce*, *Adamts9*, *Nfil3*, *Ndst1*, *Pdlim1* and *Icam1*. In addition, micro RNAs that target significant numbers of genes changed in expression in *db/db* GEC were found, including for example mir-26a, which is a potential regulator of 18 of the 267 genes. For complete lists of genes associated with specific biological processes and functions, as well as targets of transcription factors and microRNAs see Supplementary Data S11.

### The top 22 genes

**Figure 5:**
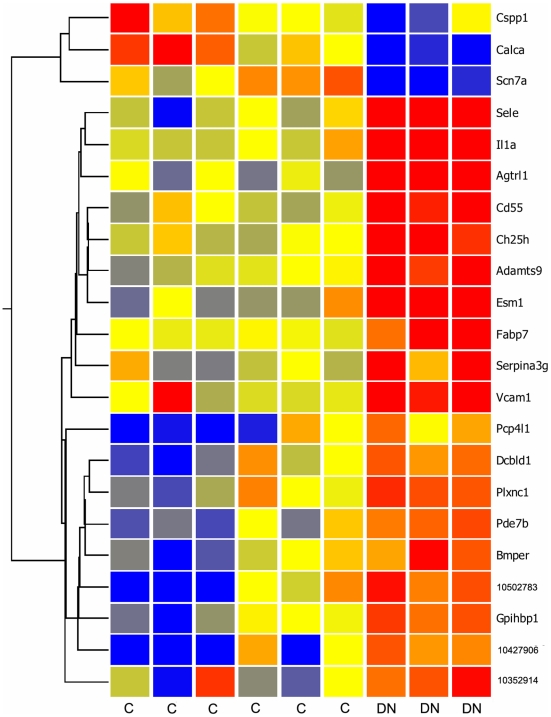
Heat Map of twenty two genes showing the greatest change in expression in glomerulus endothelial cells of diabetic nephropathy *db/db* mice. Red indicates high level of expression, and blue low expression, with yellow intermediate. **C**, control samples. **DN**, diabetic nephropathy *db/db* mice. Nineteen of the genes were up regulated in *db/db* mice, and three genes were down regulated.

A more stringent screen of the data, requiring at least three fold change in expression in *db/db* GEC, reduced the list of genes to twenty two (Fig. 5). The observed differences in expression levels were highly reproducible. Of interest, several of the genes on this list have been previously shown to be endothelial cell type specific and/or connected to the diabetic nephropathy disease process, in either a protective or pathologenic role. Some of these genes, and others with smaller fold change yet of particular interest, are described below.

### Cell adhesion molecules

Macrophages appear to play a key role in the pathogenesis of diabetic nephropathy [46], [47]. In particular, in the *db/db* mouse glomerular macrophage accumulation was previously shown to correlate well with the progression of glomerular damage [48]. Cell adhesion molecules expressed by endothelial cells can contribute to the pathogenesis of diabetic nephropathy by promoting the binding and infiltration of leukocytes. We observed elevated *Icam*, and *Vcam1* in the DB glomerular endothelial cells. This likely contributes to diabetic nephropathy pathogenesis since *Icam1* deficient mice have been reported resistant to diabetes induced renal injury [49], as a result of suppressed macrophage infiltration. It has also been shown that *db/db* mice with *Icam* deficiency have significantly reduced level of albuminuria, fewer glomerular and interstitial macrophages, and less glomerular hypetrophy, hypercellularity and tubular damage [50]. Two other cell adhesion molecules, p-selectin and e-selectin, also involved in leukocyte recruitment, have been reported elevated in the endothelial cells of patients with diabetic nephropathy [51], [52]. We observed an approximate two fold increase in *p-selectin* transcript levels in the endothelial cells of the *db/db* mice, and a nine fold elevation for *e-selectin*.

### BMP

One of the hallmarks of diabetic nephropathy is a reduction in the number of podocytes and expression of podocyte markers [53]. Decreased BMP signaling has been proposed to play a key role in podocyte injury and loss. In particular, in diabetic nephropathy podocytes produce connective tissue growth factor (CTGF), an inhibitor of BMP signaling [54]. Furthermore, BMP7 has been shown to provide protection against diabetic nephropathy in streptozotocin treated rats [55] and mice [56], [57].

The microarray data showed significant expression of *Bmp6* in the normal GECs, with high raw expression values of over 1000. Furthermore, the expression level was elevated about 1.6 fold in the *db/db* mouse (P = 0.03). This increased BMP expression might afford the podocytes some level of protection.

Another gene showing significant up regulation (3.4 fold) in the *db/db* endothelial cells was BMP binding endothelial regulator, *Bmper*, which is a secreted modulator of BMP signaling [58]. It is expressed in endothelial cells, as the name suggests, and can bind BMP2, BMP4 and BMP6 [58]. In the developing mouse BMPER appears to have primarily a “pro-BMP” function, with the knockout mouse showing developmental defects resembling those of BMP knockouts, including a smaller kidney with reduced nephron counts [59]. The elevated BMPER expression of the diabetic nephropathy GECs, combined with the increased Bmp6 expression, would be predicted to promote health and survival of the neighboring podocytes.

### TGF

While BMP expression is considered protective, increased TGFβ expression plays a central role in the pathogenesis of diabetic nephropathy. TGFβ promotes renal cell hypertrophy and extracellular matrix accumulation, two key features of diabetic nephropathy. Indeed, although perhaps extreme, it has been stated that “TGFβ has been shown to mediate virtually all of the pathologic changes of diabetic kidney disease.” [60]. Furthermore, treatment with anti TGFβ antibody has been shown to reduce or prevent hypertrophy and extracellular matrix expansion in both strptozotocin-induced diabetic mice [61], and *db/db* mice [62].

We observed a 1.5 fold increase of *TGFβ1* expression in *db/db* endothelial cells, with raw expression levels of around 375 in wild type (uncorrected P = 0.03) in GEC, while there was no significant change in *TGFβ 2* or *3*. Of interest, we also observed a modest upregulation (1.4 fold, uncorrected P =  0.006) of Smad3, a mediator of TGFβ signaling, suggesting a possible autocrine effect.

### Thrombomodulin

Glomerular endothelial cells express thrombomodulin, a receptor for thrombin. The binding of thrombin to thrombomodulin creates a complex that activates protein C, which in turn has anti-inflammatory and anti-apoptotic protective properties. Isermann et al (2007) reported that mice with streptozotocin induced diabetes showed reduced glomerular endothelial cell expression of thrombomodulin [12]. Further, they showed that genetic reduction of thrombomodulin function resulted in more severe proteinuria and extracellular matrix accumulation, while transgene expression of activated protein C protected against diabetic nephropathy. Consistent with these findings we observed high expression levels of thrombomodulin in wild type glomerular endothelial cells, with raw expression levels of about 1900, and a 1.5 fold down-regulation of expression in the *db/db* mice. This could in part account for reduced levels of activated protein C observed in diabetic nephropathy, and could contribute to pathogenesis.

### *Apelin* and *APJ*

The gene showing the second greatest fold change (7.8 fold up regulated) in the *db/db* glomerular endothelium was *Agtrl1*, angiotensin receptor like 1, also known as the APJ receptor. Apelin is an ang II homologue and is the ligand for the G-coupled receptor APJ.

*APJ* mutant mice show an enhanced vasopressor response to angiotensin II, suggesting that apelin-APJ counteracts renin-angiotensin [63]. AngII has been thought an important mediator of diabetic kidney damage, promoting vascular contraction, stimulating growth factor, cytokine and reactive oxygen synthesis (ROS) synthesis through the AT1 receptor. APJ has been localized to both vascular smooth muscle and endothelial cells [64], [65]. In particular apelin-APJ has been shown to play a role in renal hemodynamics, with apelin having a vasodilator effect that reverses that of AngII in both afferent and efferent arterioles [65], [66]. In addition, the apelin mechanism was shown to require endothelial APJ, to be mediated through NOS, and to result in calcium concentration drops in arteriole smooth muscle [65], similar to what was previously shown as a role for apelin in the regulation of aortic vascular tone [67].

In the *db/db* endothelial cells *APJ* raw expression levels were elevated about eight fold, to an extremely robust 3000. In some cases the apelin-APJ pathway has been reported to be autocrine in nature [68]. We observed low level apelin expression in wild type glomerular endothelial cells, with raw expression levels of about 150, which was unchanged in *db/db* mice. While the cardiac endothelium appears to be the major source of apelin [69], these results show that it is not the exclusive source.

The overwhelming body of data argues that apelin-APJ provides beneficial effects. APJ expression was previously shown reduced in renal arteries of *db/db* mice by western blot [66], but we show here, by microarray analysis of purified glomerular endothelial cells, and by immunohistochemistry of glomeruli (see below), that there is actually a considerably elevated level of APJ specifically within the endothelial cells of the glomerulus.

Apelin could be considered a potential therapeutic for diabetic nephropathy, reversing AngII effects. In addition it might be possible to identify a small molecule agonist of the APJ receptor, which could also provide therapeutic benefit.

Apelin-APJ has been shown to be a regulator of nitric oxide (NO) synthesis [70], with both RNA and protein levels of NO elevated in apelin treated rat aortas [71]. Altered NO levels in turn have been strongly associated with diabetic nephropathy, although the many reports do not provide a completely consistent clarification of the pathogenic and/or protective roles of NO [72]. Of interest we observed no statistically significant change in expression levels of nitrogen oxide synthase 1 (NOS1) (neuronal), NOS2 (inducible) or NOS3 (endothelial) in the *db/db* glomerular endothelial cells. Nevertheless, we cannot rule out the possibility that APJ could be driving increased NO production through altered NOS phosphorylation [70].

### *CD55*

*CD55* showed a very robust five fold elevation of expression in the endothelial cells of *db/db* mice, going from a significant raw expression level of 230 to over a thousand. CD55 is normally expressed on essentially all cells that come into contact with plasma complement proteins, and affords a protective effect. It interrupts the complement cascade and thereby helps to prevent complement mediated cell injury in inflammation. It is up regulated by a number of cytokines, including Il1a, by VEGF and by thrombin. Its increased expression in *db/db* endothelial cells likely provides a shielding consequence.

### *Esm1*

*Esm1* (endothelial specific molecule 1) showed an up regulation of about five fold in *db/db* endothelial cells, very similar to that observed for *CD55*. Also, like *CD55*, it has been shown to be up regulated by cytokines [73]. Of interest this elevated *Esm1* expression does not appear to be restricted to the kidney, with a similar 4 fold increase previously reported in the diaphragm of *db/db* mice [74]. ESM1 has been implicated in lymphangiogenesis and is potently induced by VEGF-A and VEGF-C [75]. ESM1 is a proteoglycan secreted by endothelial cells, and although its precise role is not known it has been proposed to inhibit the interaction between ICAM-1 and the LFA-1 integrin on lymphocytes and monocytes, thereby blocking cell adhesion [76].

### *Adamts9*

ADAMTS9 is a secreted disintegrin and metalloprotease carrying 15 thrombospondin type 1 repeats. The *Adamts9* gene is unusual in that the Affymetrix Mouse Gene 1.0 ST array used in this study carries six independent probesets, with a total of over 150 independent 25mer oligonucleotide probes assaying expression. All six probesets showed a statistically significant up regulation in *db/db* glomerular endothelial cells, with an average fold change of 3.8. Members of the ADAMTS family have previously been shown to be induced by inflammatory cytokines and TGFbeta1 [76], [77]. The elevated *Adamts9* expression could provide a disintegrin anticoagulant function, and it could help counteract extracellular matrix accumulation, both protective functions. But it could also contribute to glomerular basement membrane breakdown and/or effacement of podocytes, both pathologic functions. Of particular interest, ADAMTS9 has been shown to have anti-angiogenic activity, with *Adamts9* +/− heterozygous mice showing spontaneous corneal neovascularization [78]. Because of the importance of neovasculariztion in diabetic nephropathy this suggests that *Adamts9* elevated expression provides a protective function.

### *Fabp7, Gpihbp1*

*Fatty acid binding protein 7* (*Fabp7*) expression showed a four fold up regulation in *db/db* GECs. It is interesting to note that *Fabp7* was previously found expressed in a high percentage of clear renal cell carcinomas [79]. Perhaps related, *Gpihbp1* expression was also up regulated about 4 fold. It has previously been shown to encode an endothelial protein that plays a critical role in the lipolytic processing of chylomicrons [80].

### *Serpina3g*

The *Serpina3g* gene encodes a serine protease inhibitor and has been shown to provide protective effect against caspase independent cell death [81]. It is interesting to note that while serpina3g expression was elevated 4.3 fold in the *db/db* mouse, its expression level in the wild type mouse was relatively low. *Serpine2*, another serine protease inhibitor, showed only a 2.5 fold increase in *db/db* mice, but basal levels in wild type mice were higher, and as a result so was the *db/db* expression level.

### *Pde7b*

As mentioned previously, one of the most significantly altered molecular functions in the *db/db* GECs was phophodiesterase activity. *Pde7b* encodes a cAMP specific phosphodiesterase and was upregulated 3.3 fold in the *db/db* glomerulus endothelial cells. Two other cAMP specific phosphodiesterase genes, *Pde4b* and *Pde4d*, are also upregulated, to a lesser extent, about 1.5 fold. Elevated expression of these genes would work to drive down levels of cAMP, which could contribute to a breakdown of endothelial barrier properties, and modify responses to inflammatory mediators [82], [83].

### Validation

**Figure 6:**
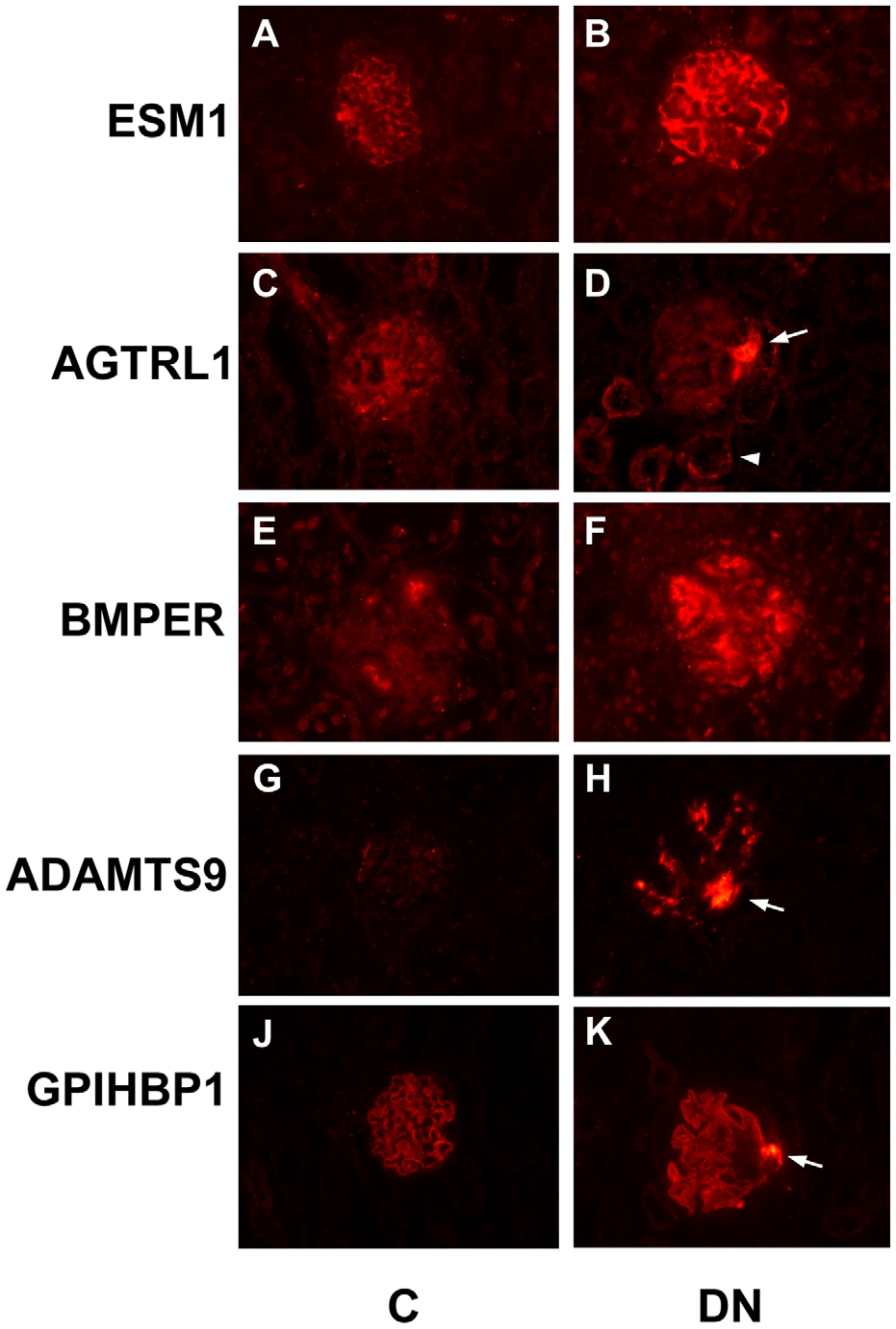
Immunostain validation of gene expression differences in *db/db* glomerulus. Protein levels were examined in C (control) kidneys, panels A, C, E, G and K, as well as DN (diabetic nephropathy) kidneys, panels B, D, F, H, and K. Elevated expression was observed in each case. Arrows in panels D, H and K show striking signal in the vascular pole region for AGTRL1, ADAMTS9 and GPIHBP1. Arrowhead in panel D marks elevated AGTRL1 expression also observed in tubules.

We performed immunostains to further examine the microarray predicted changes in gene expression. The products of five genes, *Esm1*, *Agtrl1* (*APJ*), *Bmper*, *Adamts9* and *Gpihbp1*, with up regulation in the *db/db* GECs were selected. As shown in Fig. 6, the proteins encoded by these five genes all showed significant increases in abundance in the *db/db* glomerulus. It is interesting to note that four of these proteins (ESM1, BMPR, ADAMTS9, GPIHPB1) can be secreted, so their final localizations may not necessarily coincide with the endothelial compartment. In each case, however, we observed consistent elevated expression in the *db/db* glomeruli.

It is also interesting to note that for three of the proteins, AGTRL1, ADAMTS9 and GPIHBP1, there was a dramatic increase at the vascular pole, as marked by the arrows in Fig. 6 panels D, H and K. This was highly reproducible, and not observed in the control glomeruli from non *db/db* mice. This correlates well with long standing observations demonstrating neovascularization at the vascular pole of diabetic nephropathy glomeruli [84], [85], [86], [87]. This neovascularization has been found even in diabetic patients during the first two years of disease, indicating that it is an early event [84]. Our combined microarray and immunostaining results suggest that this is a key region for endothelial gene expression changes in diabetic nephropathy.

### Summary

In summary, we defined the gene expression profiles of the endothelial cells of the kidney during development, in distinct adult compartments, and in the glomeruli of the *db/db* mouse, which represents an important animal model for human diabetic nephropathy. The results provide a comprehensive and quantitative analysis of gene use in these multiple endothelial cell populations. They define all of the growth factors, receptors and transcription factors expressed, and begin to explain the diverse properties of these divergent endothelial cell types.

The observed gene expression changes in the glomerular endothelial cells of the *db/db* mouse are perhaps of particular interest. Diabetic nephropathy is a devastating disease that occurs in approximately one third of diabetic patients [88]. A number of important previous studies have used microarrays to begin to dissect out the molecular mechanisms driving pathogenesis. Early pioneering work used whole kidneys from *db/db* or streptozotocin treated mice [89], [90], [91], and in two cases isolated glomeruli from human samples [92], [93]. In this report we were able to focus the analysis to glomerular endothelial cells, to better define the gene expression changes that occur in this precise compartment. The results identified 267 genes with greater than 1.5 fold change in gene expression, with molecular processes and biological functions including phosphodiesterase activity and cell adhesion. In addition 22 genes showed a greater than three fold change in expression, many of which could be related to either pathogenic or protective roles in the disease process. These results give a deeper understanding of diabetic nephropathy, and provide a valuable resource to facilitate further investigations.

## Material and Methods

### Endothelial cell purification

Endothelial cells were purified from *Tie2-GFP* transgenic mice using a previously described fluorescent activated cell sorting procedure [17] (http://www.gudmap.org/Research/Protocols/Potter.html). In brief, cells were quickly dissociated by a combination of enzymatic digestion and trituration. For the isolation of glomerular endothelial cells the glomeruli were first purified using a sieving procedure (see below).

### RNA Purification, Target Amplification, and Microarray Hybridization

RNA was purified using a Qiagen RNeasy Micro kits and used for a single round or RiboSpia target amplification using the Nugen WT-Pico kit according to Nugen protocols. Hybridization, washing and scanning of Affymetrix Mouse Gene 1.0 ST arrays were performed according to standard Affymetrix procedures.

### Microarray data analysis

Microarray data was analyzed as previously described [17], primarily using GeneSpring software, version GX 11.0. Other software used included ToppGene and DAVID. A standard analysis used either ANOVA for multiple sample types, or an unpaired t-test for two sample type comparisons. We removed probsets with low raw expression levels, requiring at least 100 in three samples. Probesets with low expression are particularly prone to artifact difference calls. We then used a combination of FDR, P value and fold change to adjust the stringency of the screen, with specific criteria described for each example in the text.

### Isolation of glomeruli

Kidneys were isolated in ice-cold PBS, bisected, and the medullary region was removed. The remaining cortical region was minced into very small pieces and digested with 1.0% collagenase at 37°C for 15 min, followed by vigorous trituration. An equal volume of ice-cold 5% FBS/PBS was added and the mixture was filtered through a 100 µm mesh. The flow-through was collected, triturated vigorously and filtered through a 40 µm mesh. The glomeruli trapped on the filter were isolated by turning the filter over and rinsing with ice-cold 0.1% FBS/PBS. The collected glomeruli were vigorously triturated and re-filtered through 40 µM mesh. This process was repeated three times. After the final collection, the glomeruli were pelleted, washed with ice-cold PBS and resuspended in 0.5% trypsin. The glomeruli were incubated at 37°C for 10–15 min. During incubation, the glomeruli were triturated every five min. A portion of the sample was observed under a microscope to monitor cell dissociation. After incubation, ice-cold 5% FBS/PBS was added, the cells were pelleted, washed with ice-cold PBS and filtered before FACS analysis.

### Immunoflurescence

Kidneys were isolated in ice-cold PBS, fixed overnight in 4% PFA/PBS, and then cryo-preserved at 4°C in 30% sucrose overnight. Tissue was frozen in OCT (Tissue-Tek) for cryo-sectioning. Slide mounted sections were incubated in PBS for 5 min, and blocked for 60 min in PBS containing 10% donkey serum and 0.25% Triton X-100. The sections were washed 3X in PBS containing 1% donkey serum and 0.25% Triton X-100 (PBST). The sections were incubated in PBST containing primary antibody overnight at room temperature. After incubation, the sections were washed in PBST 3 times, secondary antibody in PBST was added, and incubated for 3 hours at room temperature. Sections were washed 3X in PBST for 5 min, coverslipped in Vectashield (Vector Labs) and imaged on a Zeiss AX10 microscope (Zeiss). All primary antibodies in this study were obtained from Santa Cruz Biotechnology, Inc. (Santa Cruz CA.) and secondary antibodies were obtained from Invitrogen (Carlsbad, CA). All dilutions used were according to manufacturers recommendations.

### Ethics Statement

All mouse experiments were conducted in an AAALACS approved animal facility in accordance with the Cincinnati Children's Hospital Medical Center Institutional Animal Care and Use Committee policies, approved protocol number OD02013.

Microarray data is available at the genitourinary development atlas project web site (GUDMAP.ORG), and also through GEO (GSE21324, GSE20004, GSE 11232, GSE 22561, GSE 22464, GSE 20991). Experiments were conducted in a MIAME compliant manner.

## Supporting Information

Supplementary Data S1 Differentially expressed endothelial genes. 786 genes with most distinct expression patterns across all endothelial cell samples.
(0.15 MB XLS)

Supplementary Data S2 Embryonic endothelia versus adult endothelia elevated expression genes. 340 genes with elevated expression in embryonic endothelia compared to adult endothelia.
(0.09 MB XLSX)

Supplementary Data S3 Functional analysis of genes with elevated embryonic endothelia expression, compared to adult endothelia. ToppGene functional analysis of list of genes with elevated expression in embryonic endothelia vs adult endothelia, showing the overwhelming difference is related to cell division.
(1.08 MB XLS)

Supplementary Data S4 207 genes with elevated expression in embryonic endothelia compared to other kidney embryonic compartments.
(0.02 MB XLS)

Supplementary Data S5 DAVID functional groupings of the 207 embryonic endothelia elevated expression genes.
(0.07 MB XLS)

Supplementary Data S6 ToppGene functional analysis of the 207 genes with elevated expression in embryonic endothelia, compared to other developing kidney compartments.
(1.62 MB XLS)

Supplementary Data S7 List of twelve genes with over 4 fold elevated expression in glomerular endothelial cells.
(0.04 MB XLS)

Supplementary Data S8 List of fourteen genes with over 5 fold elevated expression in medullary endothelial cells.
(0.04 MB XLS)

Supplementary Data S9 List of genes with elevated expression in cortical endothelial cells.
(0.06 MB XLS)

Supplementary Data S10 List of 267 genes with over 1.5 fold change in gene expression level in db/db glomerular endothelial cells, compared to wild type.
(0.02 MB XLS)

Supplementary Data S11 ToppGene functional analysis of genes with changed expression in db/db glomerular endothelial cells compared to wild type.
(1.76 MB XLS)
